# In silico polypharmacology: retrospective recognition vs. rational design

**DOI:** 10.1186/1758-2946-6-S1-O25

**Published:** 2014-03-11

**Authors:** Ewgenij Proschak

**Affiliations:** 1 Institute of Pharmaceutical Chemistry, Goethe-University of Frankfurt, Frankfurt, D-60438 , Germany

## 

The „one drug – one target – one disease“ paradigm in drug discovery has been reconsidered during the last decade. This paradigm change was mainly caused by high attrition rates in drug approvals due to toxicity and lack of efficacy. Computational techniques play an important role in prediction and recognition of novel targets for approved drugs. We will discuss two machine learning approaches – self organizing maps and inverse distance weighting – for polypharmacological profiling of bioactive compounds, exemplified by two prospective studies [1,2].

While the recognition of occasional polypharmacological behavior is an established task, the rational design of multitarget ligands remains challenging. Dual or multi-target ligands have several advantages compared with selective compounds, including improved efficacy and more simple pharmacokinetic and pharmacodynamic properties in comparison to the combination of several drugs. In this context we present two in silico approaches to design dual inhibitors of 5-lipoxygenase (5-LO) and soluble epoxide hydrolase (sEH). The first study contains the discovery of a benzimidazole-based dual 5-LO/sEH inhibitor by means of in silico screening [3]. The strategy of the virtual screening protocol was an exhaustive pairwise evaluation of pharmacophore models for both targets to obtain a dual-target pharmacophore model. Our second study deals with the development of a fragment based strategy for dual-target drug discovery. Here, we applied a modified self-organizing map algorithm for in silico recognition of molecular fragments binding both targets. The predicted properties were confirmed by complementary screening techniques: STD-NMR and enzyme assay. The enlargement of the fragment hit led to submicromolar dual target inhibitor of sEH and 5-LO.[4]
